# Ventilatory oscillations at exercise: effects of hyperoxia, hypercapnia, and acetazolamide

**DOI:** 10.14814/phy2.12446

**Published:** 2015-06-24

**Authors:** Eric Hermand, François J Lhuissier, Julie Larribaut, Aurélien Pichon, Jean-Paul Richalet

**Affiliations:** 1 Université Paris 13, Sorbonne Paris Cité, Laboratoire “Hypoxie et poumon”Bobigny, France; 2 Assistance Publique-Hôpitaux de Paris, Hôpital Avicenne, Service de Physiologie, explorations fonctionnelles et médecine du sportBobigny, France

**Keywords:** Acetazolamide, exercise, hypercapnia, hyperoxia, hypoxia, periodic breathing, ventilatory oscillations

## Abstract

Periodic breathing has been found in patients with heart failure and sleep apneas, and in healthy subjects in hypoxia, during sleep and wakefulness, at rest and, recently, at exercise. To unravel the cardiorespiratory parameters liable to modulate the amplitude and period of ventilatory oscillations, 26 healthy subjects were tested under physiological (exercise) and environmental (hypoxia, hyperoxia, hyperoxic hypercapnia) stresses, and under acetazolamide (ACZ) treatment. A fast Fourier transform spectral analysis of breath-by-breath ventilation 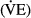 evidenced an increase in 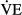 peak power under hypercapnia (vs. normoxia and hyperoxia, *P *<* *0.001) and a decrease under ACZ (vs. placebo, *P *<* *0.001), whereas it was not modified in hyperoxia. 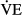 period was shortened by exercise in all conditions (vs. rest, *P *<* *0.01) and by hypercapnia (vs. normoxia, *P *<* *0.05) but remained unchanged under ACZ (vs. placebo). 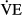 peak power was positively related to cardiac output (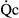) and 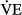 in hyperoxia (*P *<* *0.01), in hypercapnia (*P *<* *0.001) and under ACZ (*P *<* *0.001). 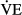 period was negatively related to 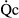 and 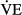 in hyperoxia (*P *<* *0.01 and *P *<* *0.001, respectively), in hypercapnia (*P *<* *0.05 and *P *<* *0.01, respectively) and under ACZ (*P *<* *0.05 and *P *<* *0.01, respectively). Total respiratory cycle time was the main factor responsible for changes in 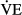 period. In conclusion, exercise, hypoxia, and hypercapnia increase ventilatory oscillations by increasing 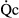 and 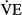, whereas ACZ decreases ventilatory instability in part by a contrasting action on O_2_ and CO_2_ sensing. An intrinsic oscillator might modulate ventilation through a complex system where peripheral chemoreflex would play a key role.

## Introduction

In most usual circumstances, human ventilation adapts to the demand of the organism around a steady-state value. However, its control is known to be challenged under various specific conditions. For example, patients suffering from chronic heart failure (CHF) or apnea syndrome (central, obstructive, or mixed) show breathing pattern marked by large oscillations with a period of around 1 min at rest, awake or asleep, and during exercise. Oscillations are also present in healthy subjects at high altitude during sleep (Berssenbrugge et al. 1983; Ainslie et al. 2013), in wakeful state (Waggener et al. 1984; Fan et al. 2012) and in patients suffering from chronic mountain sickness (Richalet et al. 2005). Several factors are involved in breathing instability. Reduced blood flow and exacerbated chemosensitivity are common in CHF at rest (Pinna et al. 2000) and during exercise (Agostoni 2008; Dhakal et al. 2012). Obstructive sleep apneas (OSA) are associated with anatomical factors inducing airway obstruction and decreased central respiratory drive to genioglossus muscle during sleep (Dempsey et al. 2010). These factors may disturb the respiratory control loop and impair the ability of the system to effectively control ventilation.

In awake subjects, only one observation of ventilatory oscillations during exercise has been reported at high altitude (Garde et al. 2012). We recently described this phenomenon, with a period of around 11 sec, in subjects exercising in mild hypoxia (Hermand et al. 2015). The analysis of cardiorespiratory parameters has highlighted some factors that may have an impact on the period and amplitude of ventilatory oscillations: unlike CHF patients whose severity of periodic breathing is related to reduced cardiac output, the amplitude of oscillations in healthy subjects was positively correlated with cardiac output and ventilation during exercise in hypoxia, whereas their period was shorter during exercise as compared to rest (Hermand et al. 2015).

Peripheral and central chemoreceptors, respectively, inform respiratory centers about O_2_ and CO_2_ status. The response to hypoxia is mainly due to peripheral chemoreceptors (Lahiri et al. 2006; Kumar and Prabhakar 2012), whereas the level of arterial or interstitial partial pressure of CO_2_ (Pco_2_) is detected by central chemoreceptors, with gain adjustments from peripheral chemoreceptors (Lahiri and Forster 2003; Blain et al. 2010), although these interactions have been debated in humans (Duffin and Mateika 2013). We suggested that peripheral chemoreceptors play an important role in the genesis of ventilatory oscillations, more pronounced in subjects with a higher hypoxic ventilatory response, thus revealing the unstable nature of the ventilatory control system under physiological and environmental stresses. The degree of ventilatory instability is linked to the loop gain, which is the product of the controller gain, that is, the ventilatory response to hypoxia and/or hypercapnia, and the plant gain, that is, the effect of a ventilation variation on arterial Pco_2_ (Paco_2_) and/or arterial Po_2_ (Pao_2_). The higher the gain, the more unstable the system (Cherniack 2005; Burgess 2012). Therefore, as in CHF, a greater sensitivity of central and peripheral respiratory drive to CO_2_ and O_2_, through greater hypoxic and hypercapnic ventilatory responses (Solin et al. 2000; Giannoni et al. 2009; Maestri et al. 2013) would lead to more pronounced oscillations of ventilation (Francis et al. 2000; Basner 2011).

Various interventions are available to modulate this breathing instability. Occasional CO_2_ inhalation during sleep considerably diminishes apnea occurrences in Cheynes-Stokes respiration (CSR) by keeping CO_2_ arterial pressure above apneic threshold (Andreas et al. 1998; Lorenzi-Filho et al. 1999). Hyperoxia has more conflicting effects: it reduces the hypoxemia involved in the mechanisms of periodic breathing, and therefore improves heart condition, physical performance, and apnea/hypopnea index in CSR patients (Andreas et al. 1996; Franklin et al. 1997), but does not abolish ventilatory oscillations in OSA (Gold et al. 1985) and CHF patients (Ponikowski et al. 1999). Conversely, at high altitude, O_2_ therapy significantly reduces sleep apneas and periodic breathing, and improves sleep quality in normal subjects (McElroy et al. 2000; Moraga et al. 2014).

Pharmacological interventions are also available for breathing disorders. Carbonic anhydrase inhibitors are proven to be effective treatment for acute (Swenson et al. 1991) and chronic (Richalet et al. 2005) mountain sickness, as well as for ventilatory disorders in healthy subjects at high altitude (Swenson et al. 1991; Fischer et al. 2004; Ainslie et al. 2013). They are also experimentally used to treat periodic breathing in CSR, CHF, and OSA patients (Edwards et al. 2012; Apostolo et al. 2014; Javaheri et al. 2014). Their complex action is still to be fully understood, and involves several contradictory mechanisms, associating central stimulation, and reduction in peripheral chemoreceptors activity (Swenson 1998; Teppema 2014). Through a greater stimulation of central chemoreceptors by CO_2_, acetazolamide (ACZ) elevates ventilation level and O_2_ saturation in patients suffering from apneas and in healthy subjects at high altitude, and considerably reduces occurrence and length of apnea episodes. ACZ also increases ventilatory response to CO_2_ in patients with chronic mountain sickness (Rivera-Ch et al. 2008). A similar effect on ventilatory oscillations was observed in awake CHF patients at rest and during exercise (Fontana et al. 2011; Apostolo et al. 2014). Similarly, the role of ACZ-induced changes in controller gain and plant gain are still debated (Burgess 2012; Edwards et al. 2012).

Until now, the phenomenon of ventilatory oscillations was well described mostly in patients and in sojourners at high altitude during sleep. Recently, a similar pattern was unveiled in normal subjects during exercise in hypoxia (Hermand et al. 2015). Since the period of oscillations observed in this preliminary study is much shorter than in patients, and as the intensity of oscillations is associated with high cardiac output (exercise) contrary to pathological conditions (CHF, CSR), this strongly suggests that the involved mechanisms are different. Therefore, the objective of this study is to unravel the mechanisms by which the ventilatory control system becomes unstable and generates oscillations in awake subjects at exercise.

In order to assess the respective role of central and peripheral chemoreceptors, respectively, sensitive to variations of CO_2_ and O_2_, we propose to submit our model of ventilatory oscillations in healthy subjects to various external constraints such as hyperoxia, hypercapnia and pharmacological treatment by ACZ.

## Subjects and Methods

### Subjects

Twenty-six healthy and nonsmoking male subjects volunteered for the study and were given complete information about the successive tests. All were in good physical condition, with a medium to high level of regular physical activity (from 2 to 10 h per week). They showed no evidence of cardiovascular or pulmonary disease. Subjects’ characteristics are presented in Table1. The protocol was approved by the Ile-de-France Ethics Committee (CPP-IDF2) and an individual written informed consent has been collected from all subjects. The study was registered as Clinical Trial reg. n°: NCT02201875.

**Table 1:** Characteristics of the subjects of the hyperoxia/hypercapnic hyperoxia and acetazolamide (ACZ) studies

	*n*	Age (years)	Body weight (kg)	Height (cm)	Maximal Aerobic Power (W)
Hyperoxia/Hypercapnic hyperoxia	13	30.0 ± 9.3	73.7 ± 9.0	174.7 ± 7.8	272.7 ± 44.2
ACZ	13	24.3 ± 3.8	71.8 ± 5.6	174.8 ± 5.6	233.3 ± 38.5

Mean ± SD. No significant difference between groups.

### Procedure

All subjects were first asked to perform a standard ramp test protocol on a cycloergometer to determine their maximal aerobic power (MAP, Table1): after a 3-min warm-up at 60 watts, power output was increased by 30-watt steps every 2 min until exhaustion.

**Figure 1:**
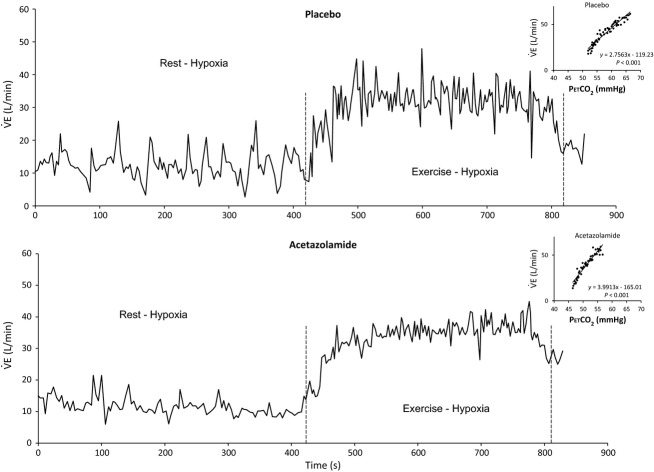
Breath-by-breath ventilation recordings in subject n° 4, under placebo (upper panel) and acetazolamide (lower panel) treatment. Inlets: ventilatory response to CO_2_ (upper right: placebo, lower right: acetazolamide).

**Figure 2:**
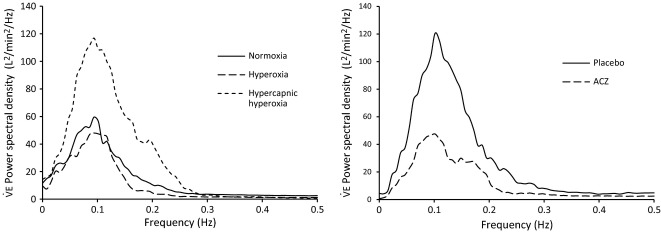
Power spectral density of the ventilation signal at exercise. Left panel: normoxia, hyperoxia, and hypercapnic hyperoxia (subject n°4). Right panel: placebo and ACZ (subject n°4) in hypoxia.

For all studies, minute ventilation (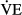, L.min^−1^) was measured through a metabograph (Vmax Encore; SensorMedics, Yorba Linda, CA). Tidal volume (VT, L), total respiratory cycle time (Ttot, sec), and inspiratory time (Ti, sec) were derived from the ventilation signal. Pulse O_2_ saturation (SpO_2_, %) was measured by transcutaneous oximetry (Nellcor N-595; Nellcor, Pleasanton, CA) on a prewarmed ear lobe. End tidal Pco_2_ (PETCO_2_, mmHg) was measured by infrared thermopile (Vmax Encore, SensorMedics). During the whole test, 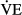, SpO_2_ and PETCO_2_ were recorded breath-by-breath (Fig.1). Data were transferred to a computer for further variability analysis. A Fast Fourier Transform (FFT) was then applied to the breath-by-breath ventilation signal, extracted from the raw data, in sequences of 128 points (one point per second) of a steady-state interval at the end of each phase of the test. This method allowed us to detect the presence of peaks in the frequency domain of the ventilation signal (Fig.2) (Hermand et al. 2015). Two main parameters were derived from the FFT: the frequency in hertz (or period in seconds) of the larger peak and its power estimated as the area under the peak at ±0.02 Hz around the peak (in L^2^·min^−2^, %^2^ and mm·Hg^2^, respectively, for 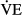, SpO_2_, and PETCO_2_ spectra). Thus, a high peak power translates into greater ventilatory oscillations. This method allowed us to precisely quantify the presence of oscillations in the signals that are not observable in the standard protocol routinely used in the hypoxic exercise test where the signals are averaged every 20 sec (Lhuissier et al. 2012; Richalet et al. 2012; Bourdillon et al. 2014; Canouï-Poitrine et al. 2014). Cardiac output (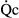, L·min^−1^) was measured using a noninvasive impedance cardiograph device (PhysioFlow PF-05; Manatec Biomedical, Paris, France) (Charloux et al. 2000; Richard et al. 2001).

The ventilatory response to CO_2_ (HCVR) was determined using a modified Read's rebreathing method (Read 1967). After breathing room air through a mouthpiece device to establish a baseline, the valve was switched to a 10-L rebreathing bag containing a mixture of 93% O_2_ and 7% CO_2_. After two deep breaths in order to speed up the gas mixing in the lungs, the subject was asked to spontaneously breathe in the bag until ventilation reached 60 L·min^−1^. Collected data were then compiled in a 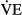-PETCO_2_ graph to calculate HCVR from the slope of the linear regression between 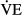 and PETCO_2_ (see inlets in Fig.1). HCVR was measured once in each subject for the hyperoxia and hypercapnic hyperoxia studies and twice (under placebo and ACZ) in the ACZ study.

### Hyperoxia and hypercapnic hyperoxia studies

Four tests were randomly conducted, in normoxia and hyperoxia (O_2_ 100%), then, at least 1 week apart, in normoxia and hypercapnic hyperoxia (O_2_ 93%, CO_2_ 7%) in 13 subjects. After 1 min rest for material habituation and cardiorespiratory parameters stabilization, subjects were first asked to keep a resting sitting position on the ergometer for 6 min, and then to pedal for 6 min at around 65 rpm pedaling cadence at an exercise intensity of 30% of MAP.

### ACZ study

In a double-blind, placebo-controlled, cross-over study, 13 subjects were asked to take either a placebo or a 250 mg ACZ pill, on morning breakfast and at midday lunch, on the day before and on tests days. Tests were performed in the afternoon following the last intake. One week at least separated the two testing days. Under each condition, subjects underwent two randomized tests: one in normoxia and one in hypoxia (normobaric simulated altitude of 3000 m). As in the hypercapnic hyperoxia studies, a 6-min rest/6-min exercise test was performed and exercise intensity was set at 30% of MAP.

#### Statistical analysis

Results are presented as mean ± standard deviation. Regarding the expected difference in mean period (3 sec), the standard deviation (2.4 sec), a level of significance at 0.05% and a statistical power at 80%, the minimum number of subjects to be included was 7 for each protocol. As spectra peak power showed a high standard deviation and a non-normal distribution, we performed a logarithmic transformation of raw data, and calculated a minimum number of subjects of 10 to include. Normality of data on each condition (rest/exercise, normoxia/hypoxia/hyperoxia/hypercapnia, placebo/ACZ) were verified by a Shapiro–Wilk normality test. In the hyperoxia and hypercapnic hyperoxia studies, two-way analyses of variance (ANOVA) with repeated measures were done (rest/exercise, normoxia/hyperoxia or normoxia/hypercapnic hyperoxia). The same subjects participated in both hyperoxic and hypercapnic hyperoxic studies. Therefore, we performed a two-way analysis of variance (rest/exercise, hyperoxia/hypercapnic hyperoxia) with repeated measures to point out the specific role of hypercapnia on cardiorespiratory parameters. In the ACZ study, a three-way analysis of variance with repeated measures was performed to evaluate the difference in period and peak power of 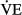, SpO_2_, and PETCO_2_ spectra between conditions (rest/exercise, normoxia/hypoxia, placebo/ACZ). A post hoc paired Student's test was then used when applicable. As exercise is associated with a concomitant variation in multiple variables, we performed a multivariate regression analysis in order to evaluate the independent influence of each cardiorespiratory variable on period and peak power of 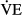, SpO_2_, and PETCO_2_ spectra. This approach, for example, will allow us to assess the specific influence of 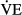 and/or 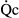 on peak power and period of 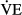.

## Results

Values of measured cardiorespiratory variables are presented in Table2.

**Table 2:** Mean values of cardiorespiratory parameters for the hyperoxia (Hox), hypercapnic hyperoxia (Hox Hcap), and acetazolamide (ACZ) studies

	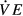 (L·min^−1^)		SpO_2_ (%)		PETCO_2_ (mmHg)		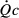 (L·min^−1^)		Ti (sec)		Ttot (sec)		VT (L)		HCVR (L·min^−1^ ·mmHg^−1^)
	Rest	Exer.	Rest	Exer.	Rest	Exer.	Rest	Exer.	Rest	Exer.	Rest	Exer.	Rest	Exer.	
**Study Hox**															
Nx	11.4 ± 1.9	33.9 ± 4.2###	96.2 ± 1.2	96.0 ± 1.4	38.7 ± 1.7	45.8 ± 2.3	5.53 ± 1.07	10.33 ± 1.81###	1.84 ± 0.62	1.40 ± 0.43	5.04 ± 1.56	3.16 ± 0.92##	0.92 ± 0.20	1.74 ± 0.41###	2.78 ± 0.86
Hox	12.5 ± 2.5	32.9 ± 4.7###	98.0 ± 0.8***	98.0 ± 0.7***	36.8 ± 2.1***	45.4 ± 3.4###	5.34 ± 0.79	9.78 ± 1.42###^,^*	1.42 ± 0.37**	1.34 ± 0.44	4.75 ± 1.65	3.28 ± 0.94##	0.93 ± 0.21	1.73 ± 0.34###	
**Study Hox Hcap**															
Nx	11.9 ± 2.0	34.2 ± 5.6###	96.6 ± 1.4	96.3 ± 1.6#	38.9 ± 2.2	45.7 ± 2.7###	5.66 ± 1.14	10.22 ± 1.47###	1.75 ± 0.51	1.39 ± 0.43##	4.65 ± 1.38	3.08 ± 0.90###	0.90 ± 0.24	1.70 ± 0.38###	
Hox Hcap	35.5 ± 8.5***, +++	72.9 ± 13.9###^,^***^,^+++	97.9 ± 0.5**	97.9 ± 0.5**	55.4 ± 2.7***^,^+++	66.3 ± 3.4###^,^***^,^+++	5.25 ± 0.93	10.76 ± 1.62###^,^*^,^+	1.37 ± 0.29**	1.04 ± 0.23###^,^***^,^++	3.36 ± 0.79***^,^++	2.29 ± 0.50###^,^***^,^+++	1.91 ± 0.42***^,^+++	2.59 ± 0.37###^,^***^,^+++	
**Study ACZ**															
Placebo															
Nx	11.6 ± 1.2	31.5 ± 4.4###	96.3 ± 1.6	96.1 ± 1.3	40.5 ± 3.1	46.9 ± 3.1###	6.18 ± 1.06	11.19 ± 1.60###	1.88 ± 0.81	1.54 ± 0.54#	4.58 ± 1.54	3.34 ± 0.99###	0.87 ± 0.28	1.71 ± 0.38###	2.46 ± 0.53
Hx	12,3 ± 1.5	33.9 ± 4.2###^,^*	91.8 ± 1.9***	86.8 ± 1.5###^,^***	40.2 ± 2.9	44.8 ± 1.8###^,^*	6.93 ± 1.17*	12.45 ± 2.35###^,^*	1.77 ± 0.61	1.46 ± 0.53	4.23 ± 1.07	3.12 ± 0.90###	0.85 ± 0.21	1.72 ± 0.38###	
ACZ															
Nx	12.3 ± 1.8	35.3 ± 4.7###^,^††	96.3 ± 1.4	96.0 ± 1.1	34.4 ± 3.5†††	40.3 ± 4.6###^,^†††	6.93 ± 1.17	12.45 ± 2.35###	1.88 ± 0.61	1.46 ± 0.53#	4.73 ± 2.20	3.19 ± 0.91##	0.93 ± 0.30	1.82 ± 0.41###	3.78 ± 1.67††
Hx	12.8 ± 1.5	36.3 ± 5.5###	92.5 ± 1.3***	88.3 ± 2.5###^,^***^,^†	34.1 ± 3.8†††	37.9 ± 4.5###^,^**^,^†††	6.52 ± 1.42**	12.08 ± 2.36###^,^***	1.89 ± 0.86	1.42 ± 0.44##	4.64 ± 1.58	3.19 ± 0.82###	0.96 ± 0.27	1.87 ± 0.34###	

Nx, normoxia

Hx, hypoxia

Exer., Exercise.

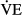
, minute ventilation

SpO_2_, pulse O_2_ saturation

PETCO_2_, end-tidal Pco_2_

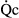
, cardiac output

Ti, inspiration time

Ttot, total respiration cycle time

VT, tidal volume

HCVR, hypercapnic ventilatory response.

Mean ± SD.

Exercise versus Rest:

# *P *<* *0.05

## *P *<* *0.01

### *P *<* *0.001.

Hypercapnic hyperoxia or hyperoxia or hypoxia versus normoxia:

* *P *<* *0.05

** *P *<* *0.01

*** *P *<* *0.001.

Hypercapnic hyperoxia versus hyperoxia:

+ *P *<* *0.05

++ *P *<* *0.01

+++ *P *<* *0.001.

ACZ versus placebo:

† *P *<* *0.05

†† *P *<* *0.01

††† *P *<* *0.001.

### Effect of hyperoxia

#### Effect of exercise versus rest

**Figure 3:**
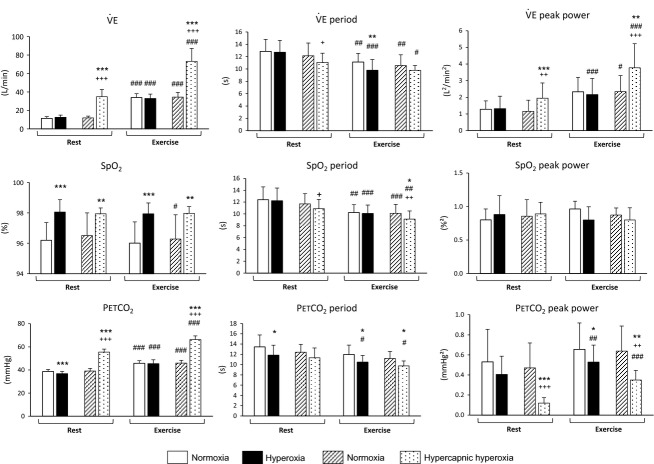
Mean (±SD) values of 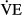, SpO_2_, and PETCO_2_ and corresponding periods and peak powers in normoxia, hyperoxia, and hypercapnic hyperoxia. Exercise versus Rest: ^#^*P *<* *0.05; ^##^*P *<* *0.01; ^###^*P *<* *0.001. Hypercapnic hyperoxia or hyperoxia versus normoxia: *P <* *0.05; **P <* *0.01; ***P <* *0.001. Hypercapnic hyperoxia versus hyperoxia: ^+^*P *<* *0.05, ^++^*P *<* *0.01, ^+++^*P *<* *0.001.

In the two-way ANOVA, 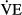 and PETCO_2_ increased with exercise (*P *<* *0.001), SpO_2_ being unchanged (Fig.3). 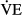 period was shorter (*P *<* *0.001) and its peak power higher (*P *<* *0.001). PETCO_2_ period decreased (*P *<* *0.001) and its peak power increased (*P *<* *0.05). SpO_2_ period was shorter (*P *<* *0.001).

#### Effect of hyperoxia versus normoxia

In the two-way ANOVA, 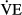 was not different in hyperoxia, whereas SpO_2_ increased (*P *<* *0.001) and *P* ETCO_2_ decreased (*P *<* *0.05). 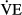 period tended to be slightly shorter (*P *=* *0.07), as well as PETCO_2_ period. PETCO_2_ peak power decreased (*P *<* *0.01).

The post hoc analysis showed that, at exercise, 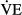 and PETCO_2_ periods were shorter in hyperoxia (vs. normoxia, *P *<* *0.01). 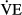 peak power remained unchanged, whereas PETCO_2_ peak power decreased significantly (*P *<* *0.05).

### Effect of hypercapnic hyperoxia

#### Effect of exercise versus rest

In the two-way ANOVA, 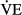 and PETCO_2_ increased with exercise (*P *<* *0.001) (Fig.3). 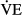 and PETCO_2_ periods were shorter (*P *<* *0.01 and *P *<* *0.001, respectively) and their peak power greater (*P *<* *0.001).

#### Effect of hypercapnic hyperoxia versus normoxia

In the two-way ANOVA, 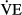, SpO_2_, and PETCO_2_ increased (*P *<* *0.001) in hypercapnic hyperoxia. 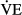 period was shorter and its peak power was greater (*P *<* *0.05 and *P *<* *0.001, respectively). PETCO_2_ period was shorter (*P *<* *0.01) and its peak power was smaller (*P *<* *0.001).

The post hoc analysis showed that, at exercise, SpO_2_ and PETCO_2_ periods decreased in hypercapnic hyperoxia (vs. normoxia, *P *<* *0.05). 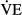 peak power was greater (vs. normoxia, *P *<* *0.01), whereas PETCO_2_ peak power was lower (vs. normoxia, *P *<* *0.01).

### Effect of hypercapnia: hypercapnic hyperoxia versus hyperoxia

A two-way ANOVA evidenced an increase in 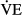 and PETCO_2_ in hypercapnic hyperoxia (vs. hyperoxia, *P *<* *0.001) (Fig.3). As expected, SpO_2_ was not significantly different between conditions. The period of ventilatory oscillations tended to be shorter in hypercapnic hyperoxia (vs. hyperoxia, *P *=* *0.054). 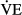 peak power was greater in hypercapnic hyperoxia (vs. hyperoxia, *P *<* *0.001). PETCO_2_ period was not modified by hypercapnia, whereas PETCO_2_ peak power decreased (*P *<* *0.001).

### Effect of ACZ

**Figure 4:**
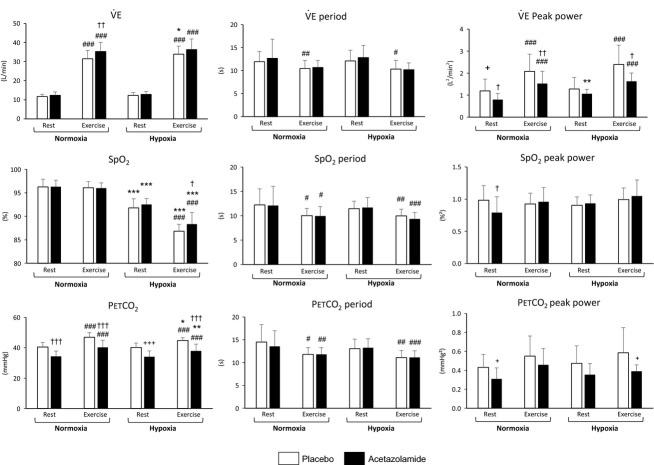
Mean (±SD) values of 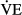, SpO_2_, and PETCO_2_ and corresponding periods and peak powers in normoxia (Nx) and hypoxia (Hx), under placebo or acetazolamide treatment. Exercise versus Rest: ^#^*P *<* *0.05; ^##^*P *<* *0.01; ^###^*P *<* *0.001. Hypoxia versus normoxia: *P <* *0.05; **P <* *0.01; ***P <* *0.001. Acetazolamide versus placebo: ^†^*P *<* *0.05, ^††^*P *<* *0.01, ^†††^*P *<* *0.001.

One subject had to stop the experimentation due to side effects of ACZ administration (headaches, nausea), so that 12 subjects performed the experiment (Fig.4).

Typical complete breath-by-breath recordings in hypoxia under placebo (upper panel) and ACZ (lower panel) are presented in Fig.1. Oscillations of 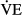 during exercise are clearly less noticeable under ACZ condition, which is confirmed by a lower peak power in the corresponding frequency spectrum of the ventilation signal (Fig.2, right).

#### Effect of exercise versus rest

During exercise, 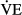 and PETCO_2_ increased (*P *<* *0.001) and SpO_2_ decreased (*P *<* *0.001). 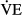, SpO_2_ and PETCO_2_ periods were shorter (*P *<* *0.001). 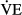 and PETCO_2_ peak powers were higher (*P *<* *0.001 and *P *<* *0.01, respectively).

#### Effect of hypoxia versus normoxia

In hypoxia, SpO_2_ and PETCO_2_ were lower (*P *<* *0.001 and *P *<* *0.01, respectively), and 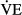 tended to be higher (*P *=* *0.053). 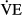, SpO_2_, and PETCO_2_ periods were not significantly different, whereas 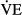 and PETCO_2_ peak power increased (*P *<* *0.001 and *P *<* *0.01, respectively).

#### Effect of ACZ versus placebo

ACZ increased 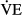 (*P *<* *0.01) and lowered PETCO_2_ (*P *<* *0.001). As expected, SpO_2_ did not change in normoxia. When considering only hypoxic condition, ACZ increased SpO_2_ (*P *<* *0.05). 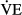, SpO_2_, and PETCO_2_ periods were not modified. 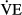 and PETCO_2_ peak powers were lower under ACZ (*P *<* *0.001). For all subjects, the average period was around 12.4 sec at rest and significantly decreased to 10.4 sec at exercise (*P *<* *0.001), without any difference between the placebo and the ACZ groups.

### Cardiorespiratory variables affecting 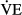 period and 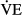 peak power

**Figure 5:**
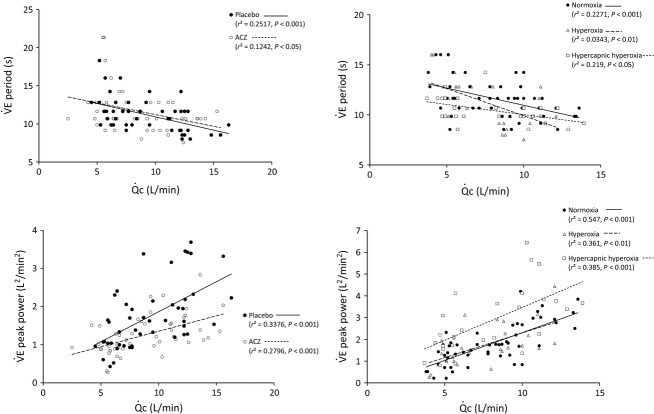
Ventilation period (upper panels) and peak power (lower panels) as a function of cardiac output (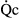). Left panels: ACZ and placebo. Right panels: normoxia, hyperoxia, and hypercapnic hyperoxia. Rest and exercise values have been pooled in all conditions. Normoxia and hypoxia values have been pooled in the ACZ versus placebo study. Slopes are significantly different between placebo and ACZ for the 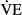 peak power versus 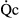 correlation (*P *<* *0.05).

**Figure 6:**
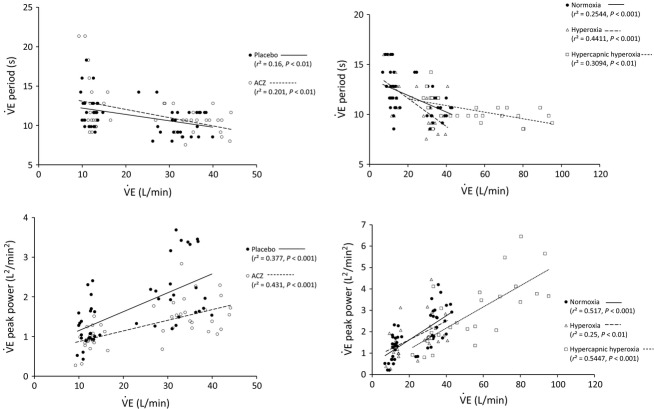
Ventilation period (upper panels) and peak power (lower panels) as a function of ventilation (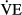). Left panels: ACZ and placebo. Right panels: normoxia, hyperoxia, and hypercapnic hyperoxia. Rest and exercise values have been pooled in all conditions. Normoxia and hypoxia values have been pooled in the ACZ versus placebo study. Slopes are significantly different between placebo and ACZ for the 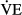 peak power versus 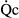 correlation (*P *<* *0.05).

**Figure 7:**
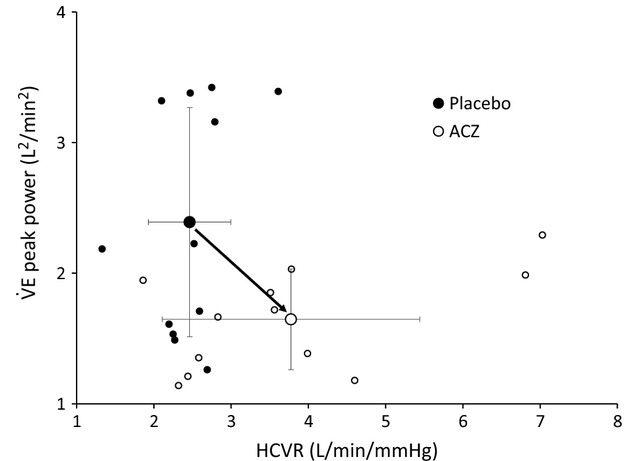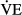 peak power as a function of HCVR for each subject under placebo and ACZ (large symbols: mean values ± SD, black dot, Placebo; white dot, ACZ). ACZ increases HCVR and reduces 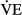 peak power, but these relationships do not reach significance (Placebo: *r*² = 0.123, *P *=* *0.26; ACZ: *r*² = 0.273, *P *=* *0.08). However, it is clear that ACZ blunts the effect of HCVR on ventilatory oscillations (arrow).

In a first univariate approach, regressions for 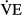 period and 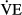 peak power, for each condition (normoxia, hyperoxia, hypercapnic hyperoxia, placebo, and ACZ), with 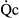 or 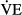 were assessed (Figs.5 and 6, respectively). In all conditions, 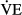 period was negatively related to 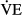 and 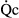 (upper panels), whereas 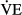 peak power was positively correlated with 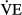 and 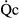 (lower panels). It is noticeable that ACZ administration significantly decreased (vs. placebo) the slope of the regression between 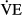 peak power and 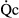 as well as 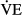 (*P *<* *0.05): the higher 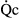 or 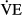, the greater the effect of ACZ (Figs.5 and 6, lower left panels). In addition, for a given level of 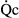, 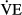 peak power was always higher in hypercapnia, the regression slopes being similar (Fig.5, lower right panel). HCVR was higher under ACZ (vs. placebo, *P *<* *0.01, Table2). However, there was no significant correlation between HCVR and the corresponding 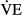 peak power, under placebo and ACZ (Fig.7), nor between HCVR placebo-ACZ difference and the corresponding 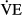 peak power placebo-ACZ difference (*P *=* *0.18). Although the linear relationship between 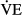 peak power and HCVR did not reach significance, Fig.7 clearly shows that for a given level of HCVR, ACZ decreases 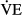 peak power.

**Figure 8:**
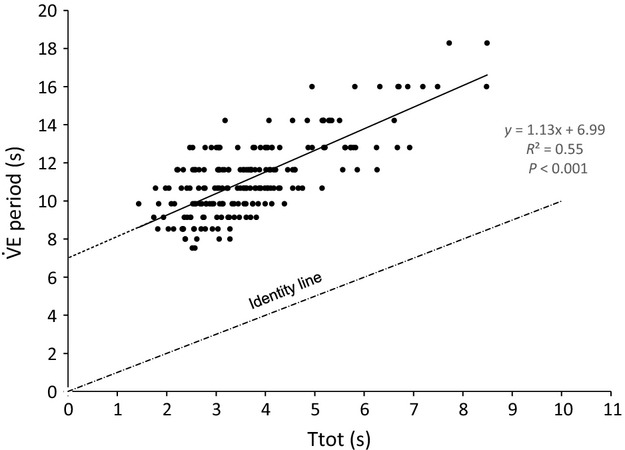
Linear regressions between 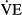 period and total respiratory cycle time (Ttot) in the ACZ study. All values (rest/exercise, normoxia/hypoxia, placebo/ACZ) have been pooled.

As several variables, such as 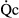, 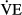 and Ttot, may vary simultaneously in response to exercise or environmental constraints, we performed a multivariate regression analysis to assess the specific role of each of these variables on ventilatory oscillations. When pooling all values obtained at rest and exercise, this analysis evidenced Ttot as the most significant factor explaining changes in 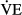 period (*P *<* *0.001), as illustrated in Fig.8. Concerning 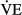 peak power, 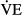 (*P *<* *0.001) and Ttot (*P *<* *0.02), but not 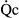 (*P *=* *0.094), were found as explaining variables.

### To summarize the main results of these three studies:

1. Hyperoxia reduced the period of 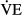 and PETCO_2_ oscillations and decreased PETCO_2_ peak power at exercise.
2. Hypercapnia increased 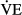 peak power and decreased 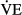 period, whereas it reduced the amplitude of PETCO_2_ oscillations.
3. ACZ blunted 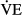 and PETCO_2_ oscillations but did not alter corresponding periods.
4. Exercise increased amplitudes and shortened periods of 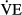 and PETCO_2_ oscillations in all conditions.

## Discussion

To our knowledge, this is the first study on ventilatory oscillations performed on healthy subjects under physiological (exercise) and environmental stress (hypoxia/hyperoxia/hypercapnia) and under pharmacological challenge (ACZ). Our previous work evidenced for the first time ventilatory oscillations in hypoxia at exercise in normal subjects, and the importance of peripheral chemoreceptors in their genesis (Hermand et al. 2015). This study reveals further factors that may modulate these oscillations and proposes some mechanistic hypotheses. Hyperoxia, which silences peripheral chemoreceptors (Sinski et al. 2014), did not modify 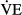 peak power and therefore 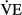 oscillations in healthy subjects, in accordance with some but not all studies in patients with mixed sleep apnea (Brack et al. 2012). We evidenced a very significant increase in 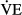 peak power in hypercapnia, conversely to what was found in central and obstructive apneas, where CO_2_ inhalation drastically reduced ventilatory oscillations (Hudgel et al. 1988; Brack et al. 2012). The effect of ACZ treatment in healthy subjects in hypoxia at exercise was similar to what was observed in subjects at high altitude (Ainslie et al. 2013) and CSR-OSA patients (Lalande et al. 2009; Brack et al. 2012): average minute ventilation was augmented and PETCO_2_ decreased, whereas ventilatory oscillations were considerably reduced at exercise in hypoxia. This confirms the blunting of loop gain by ACZ observed in OSA patients (Edwards et al. 2012). It has been reported that ACZ could impair respiratory muscle and therefore modify the breathing pattern during exercise (Gonzales and Scheuermann 2013). However, a much lower dose of ACZ was used in this study and exercise intensity was limited to 30% of MAP. Moreover, as VT and Ti were not modified by ACZ in rest or exercise conditions (Table2), we therefore assume that ACZ did not impact breathing pattern.

### Mechanisms

**Figure 9:**
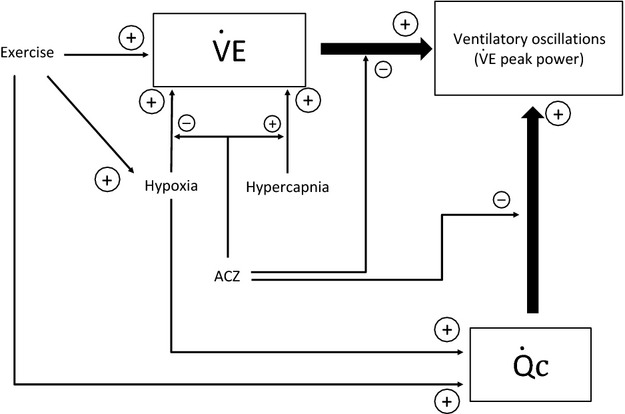
Schematic diagram of potential mechanisms involved in the genesis of ventilatory oscillations in normal subjects at exercise. Breathing instability is directly related to the intensity of 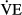 and 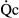. Exercise, hypoxia and hypercapnia increase 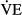 and 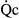, and therefore increase ventilatory oscillations. ACZ inhibits the effect of hypoxia and enhances the effect of hypercapnia on ventilation, and blunts the relation between 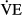 or 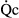, and ventilatory oscillations. The complex effects of ACZ on this regulation system are still debated (see discussion).

These results provide new insights on mechanisms involved in ventilatory oscillations in healthy subjects (Fig.9). The activity of the respiratory central pattern generator could be modulated by an oscillator, the activity of which would depend on various chemical stimuli. This oscillator would promote the instability of the system. Given that this instability seems to be directly related to the intensity of 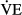 and 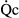, it is not surprising to observe the destabilizing effect of exercise, hypoxia and hypercapnia, which are known to increase 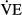 and 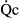. ACZ has contrasting effects: it inhibits the effect of hypoxia (Ainslie et al. 2013) and enhances the effect of hypercapnia on ventilation (Vovk et al. 2000). In this study, ACZ also blunts the relation between ventilatory oscillations and 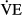 or 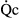 (Figs.5 and 9). Hyperoxia deactivates peripheral chemoreceptors (Sinski et al. 2014), and, to our knowledge, has no action on central chemoreceptors in humans. Although hyperoxia did not significantly decrease 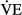 peak power, as we could expect regarding the inhibition of peripheral chemoreceptors, there is a tendency for a decrease in 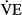 and SpO_2_ peak power at exercise and a significant decrease in PETCO_2_ peak power. Therefore, the hyperoxia-induced inhibition of carotid bodies would not be strong enough, alone, to modulate ventilatory oscillations.

CO_2_ stimulates central chemoreceptors, but is also, in a lesser extent, a determinant factor in peripheral chemoreflex activation in hypoxia (Mohan and Duffin 1997). Therefore, the noticeable increase in ventilatory oscillations in hypercapnia could be mainly attributed to the activation of central chemoreceptors, without completely ruling out a potential peripheral involvement of CO_2_ in hyperoxia.

Acetazolamide stimulates central chemoreceptors but reduces peripheral chemoreceptors activity (Ainslie et al. 2013). However, its mechanisms of action have been extensively debated (Teppema 2014). Putting the decrease in ventilatory oscillations under ACZ in perspective with the increase in these oscillations in hypoxia when peripheral chemoreceptors are stimulated, confirms that peripheral chemoreceptors play a key role in the genesis of ventilatory oscillations. It validates the hypothesis we put forward in our preliminary study showing that oscillations are enhanced in subjects with high HVR (Hermand et al. 2015). However, ventilatory oscillations are also greater under a hyperoxic hypercapnic stress, when peripheral chemoreceptors are silenced, thus implying that central chemoreceptors are also a major actor of breathing instability. This suggests that ventilatory oscillations depend on a subtle and synergic action of both central and peripheral chemoreceptors, through arterial O_2_ and CO_2_ pressure.

Moreover, we observed that, in hypercapnic hyperoxia, PETCO_2_, and 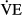 exhibited dichotomous oscillations properties: PETCO_2_ peak power was paradoxically lower in hypercapnia whereas 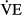 peak power was higher. CO_2_ stores being close to saturation in hypercapnia, despite a higher ventilation to flush it out, the measured expired CO_2_ level is then not subjected to oscillations as observed for 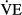, as a full CO_2_ store will damp systemic CO_2_ variations.

ACZ augments the controller gain (HCVR) but blunts the enhancing effects of controller gain on ventilatory oscillations (Fig.7). This paradoxical effect of ACZ, reducing ventilatory oscillations (i.e., loop gain) in spite of the rise of controller gain, means a very significant reduction of plant gain, evoked in Javaheri's work (Javaheri et al. 2014). While we were not able to determine the value of the plant gain, we nevertheless observed a noticeable decrease in PETCO_2_ under ACZ in all conditions (Fig.4), therefore inducing a lower CO_2_ reserve and a lower plant gain (White 2005; Javaheri et al. 2014).

Mechanisms leading to reduced ventilatory oscillations under ACZ are still debated. This may involve a third arm, the mixing gain, depending, among other factors, on cerebral blood flow which might increase the CO_2_ washout of the cerebral spinal fluid and therefore blunt ventilatory oscillations (Younes et al. 2001; Dempsey et al. 2004; Skow et al. 2014). However, both exercise (Ogoh and Ainslie 2009) and ACZ (Okazawa et al. 2001) are known to increase cerebral blood flow but have opposite effects on breathing instability (this study).

The multivariate analysis showing that the intensity of oscillations is related to 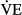 and Ttot, but insignificantly to 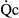, gives a clue to the apparent contradiction between our observations of greater ventilatory oscillations with increasing 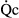 (Fig.5) and the current hypothesis of low 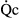 induced periodic breathing in CHF patients (Agostoni 2008).

The tight relationship between 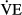 period and Ttot observed in our previous work is confirmed (Fig.8): 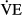 period is strongly linked to the duration of the respiratory cycle, independently of other cardiorespiratory factors such as 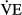 and 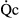. This suggests that Ttot is a determinant factor of the intrinsic oscillator. This correlation also pointed out a theoretical limit of 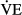 oscillation period: when Ttot tends toward zero (breathing frequency tending to infinity), 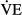 oscillation period tends to approximately 7 sec. This tight relationship between 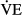 period and Ttot also suggests the involvement of an internal oscillator that would modulate the activity of the central pattern generator, responsible for the generation of respiratory rhythm in mammals (Forster et al. 2014).

In conclusion, these findings confirm the existence of a mechanism that modulates ventilation and induces breathing instability, especially during exercise in hypoxic and hypercapnic conditions. This instability, unraveled in healthy awake subjects after a brisk challenge, depends on a complex regulation system involving O_2_ and CO_2_ sensing where both peripheral (high HVR and effect of ACZ) and central (hypercapnia and tight relationship between period and Ttot) chemoreflex seem to play a key role. We expect this study to contribute to the knowledge of factors involved in sleep apneas of central and obstructive origins.

## Conflict of Interest

None declared.
